# Changes and significance of gut microbiota in children with focal epilepsy before and after treatment

**DOI:** 10.3389/fcimb.2022.965471

**Published:** 2022-11-03

**Authors:** Changci Zhou, Shuaizheng Gong, Shiting Xiang, Lijuan Liang, Xia Hu, Ruiwen Huang, Zhenyu Liao, Ye Ma, Zhenghui Xiao, Jun Qiu

**Affiliations:** ^1^ Academy of Pediatrics, Hengyang Medical School, University of South China, Hengyang, China; ^2^ Department of Hematology and Oncology, Hunan Children’s Hospital, Changsha, China; ^3^ Pediatrics Research Institute of Hunan Province, Hunan Children’s Hospital, Changsha, China; ^4^ Department of Emergency Center, Hunan Children’s Hospital, Changsha, China; ^5^ Department of Neonatology, Hunan Children’s Hospital, Changsha, China

**Keywords:** gut microbiota, epilepsy, focal onset, children, 16S rDNA gene sequencing

## Abstract

**Objective:**

To better understand the alterations in gut microbiota and metabolic pathways in children with focal epilepsy, and to further investigate the changes in the related gut microbiota and metabolic pathways in these children before and after treatment.

**Methods:**

Ten patients with newly diagnosed focal epilepsy in Hunan Children’s Hospital from April, 2020 to October, 2020 were recruited into the case group. The case group was further divided into a pre-treatment subgroup and a post-treatment subgroup. Additionally, 14 healthy children of the same age were recruited into a control group. The microbial communities were analyzed using 16s rDNA sequencing data. Metastas and LEfSe were used to identify different bacteria between and within groups. The Kyoto Encyclopedia of Genes and Genomes database was used to KEGG enrichment analysis.

**Results:**

There were significant differences in α diversity among the pre-treatment, post-treatment, and control groups. Besides, the differences in gut microbiota composition in 3 groups were identified by principal co-ordinates analysis (PCoA), which showed a similar composition of the pre-treatment and post-treatment subgroups. At the phyla level, the relative abundance of *Actinobacteria* in the pre-treatment subgroup was significantly higher than that in the control group, which decreased significantly after 3 months of treatment and showed no significant difference between the control group. In terms of the genus level, *Escherichia/Shigella*, *Streptococcus*, *Collinsella*, and *Megamonas* were enriched in the pre-treatment subgroup, while *Faecalibacterium* and *Anaerostipes* were enriched in the control group. The relative abundance of *Escherichia/Shigella*, *Streptococcus*, *Collinsella*, and *Megamonas* was reduced significantly after a three-month treatment. Despite some genera remaining significantly different between the post-treatment subgroup and control group, the number of significantly different genera decreased from 9 to 4 through treatment. Notably, we found that the carbohydrate metabolism, especially succinate, was related to focal epilepsy.

**Conclusion:**

Children with focal epilepsy compared with healthy controls were associated with the statistically significant differences in the gut microbiota and carbohydrate metabolism. The differences were reduced and the carbohydrate metabolism improved after effective treatment. Our research may provide new directions for understanding the role of gut microbiota in the pathogenesis of focal epilepsy and better alternative treatments.

## Introduction

Epilepsy is a disease of the brain defined by the propensity for an individual to have recurrent unprovoked epileptic seizures (Fisher et al., 2014). Children are at a high risk of epilepsy. In general, epilepsy is mainly divided into focal, comprehensive, and unknown seizures according to the clinical manifestations and electroencephalogram (EEG) of epileptic seizures, in which focal seizures are the most common type of epileptic seizures in children (Jiang et al., 2019). Oral antiepileptics, mainly oxcarbazepine (OXC), are recommended as the primary treatment of epilepsy according to current expert consensus on pediatric epilepsy monotherapy (Department of Neurology, Chinese Pediatric Society, Chinese Medical Association, 2015). Although research has shown that antiepileptic drugs are effective in treating children with focal epilepsy (Zhong et al., 2018), some children are resistant to antiepileptic drugs and develop intractable epilepsy. Furthermore, focal seizures can be followed by other serious seizures, such as the focal secondary bilateral tonicoclonic seizures. Uncontrolled seizures with epilepsy may lead to cognitive deficits, and permanent brain dysfunction (Dahlin and Prast-Nielsen, 2019), which seriously affect the normal growth and development of children.

Recently, gut microbiota has attracted the growing attention of researchers. Brain-gut axis is reported to be the bi-directional connection between the brain and the gastrointestinal tract, where gut microbiota is an important part (Zhu et al., 2017). Therefore, these bacteria probably react to and influence neuronal, neural and humoral, metabolic, or immune signaling underlying the gut-brain relationship. Bacteria from different parts of the gastrointestinal tract have positive effects on the growth and development of the central nervous system, such as neurogenesis, microglia maturation, and myelinization (Erny et al., 2015; Hoban et al., 2016), which are associated with human functions of cognition, emotion, and behavior (Clarke et al., 2013), as well as a variety of neurological disorders (Iannone et al., 2019). The microbiota-gut-brain axis is being considered a new direction to explain the complex characteristics of different neurological diseases (Borghi and Vignoli, 2019; Cryan et al., 2020). Moreover, there is evidence showing gut microbiota’s potential effects on epilepsy. Recent studies have hypothesized that the gut microbiota may contribute to a pro-inflammatory state, driving drug-resistant seizures (Dahlin & Prast-Nielsen, 2019; De Caro et al., 2019). A case of Crohn’s disease with a 17-year history of epilepsy was reported in the literature. The patient had about 120 seizures per year and stopped having any for nearly 20 months without any antiepileptic drugs after fecal transplant treatment (He et al., 2017). Another study found that gut microbiota in patients with drug-resistant epilepsy showed obvious changes compared with those who were drug-sensitive and healthy (Peng et al., 2018). The ketogenic diet (KD) has been recognized as an effective alternative for treating intractable epilepsy. After KD treatment, the number of seizures decreased by more than 50% in 5 cases and the cognitive and motor functions improved in 10 cases, with a significant difference in the microbial communities (Lindefeldt et al., 2019). In another study, treatment of probiotic supplementation was suggested to reduce the frequency of seizures by 50% or even more in 28.9% of patients with drug-resistant epilepsy (Gómez-Eguílaz et al., 2018). In conclusion, gut microbiota is linked to early epilepsy diagnosis (Medel-Matus et al., 2018) and clinical intervention.

Although there are increasing studies on epilepsy and gut microbiota, most of them focus on intractable epilepsy, whose effects on gut microbiota remain unclear. In particular, studies on the alterations of gut microbiota in drug-treated patients with newly diagnosed epilepsy are few. Antiepileptic drugs (Stokes et al., 2014; de Theije et al., 2014; Liu et al., 2018), as well as epilepsy types, may also have an impact on gut microbiota. The present study aims at describing the alterations in the gut microbiota between children with focal epilepsy and healthy children, and the changes in the gut microbiota of children with focal epilepsy before and after monotherapy, to provide a new direction for understanding the pathogenesis of focal epilepsy and a better alternative treatment.

## Materials and methods

### Study subjects

We enrolled 17 children admitted to the Neurology Department of Hunan Children’s Hospital (Changsha, China) from April 2020 to October 2020, with newly diagnosed focal epilepsy. The diagnostic criteria for focal epilepsy were referred to in ILAE 2017 (Fisher et al., 2017). Inclusion criteria were that the subjects were over the age of two years, that the EEG and clinical manifestations supported the diagnosis of focal epilepsy, that there was no abnormality in head MRI, and that there was no family history of epilepsy. We collected fecal samples from subjects before and three months after the only OXC treatment. Exclusion criteria were chronic or acute intestinal diseases, special diets, and treatment with antibiotics or probiotics within the first two weeks of the two collection time points. Finally, children who did not regularly take antiepileptic drugs, did not respond to treatment, or were lost to follow-up were excluded. After filtering out one case not responding to treatment and six cases lost to follow-up, data of ten children were remained for further study.

**Figure 1:**
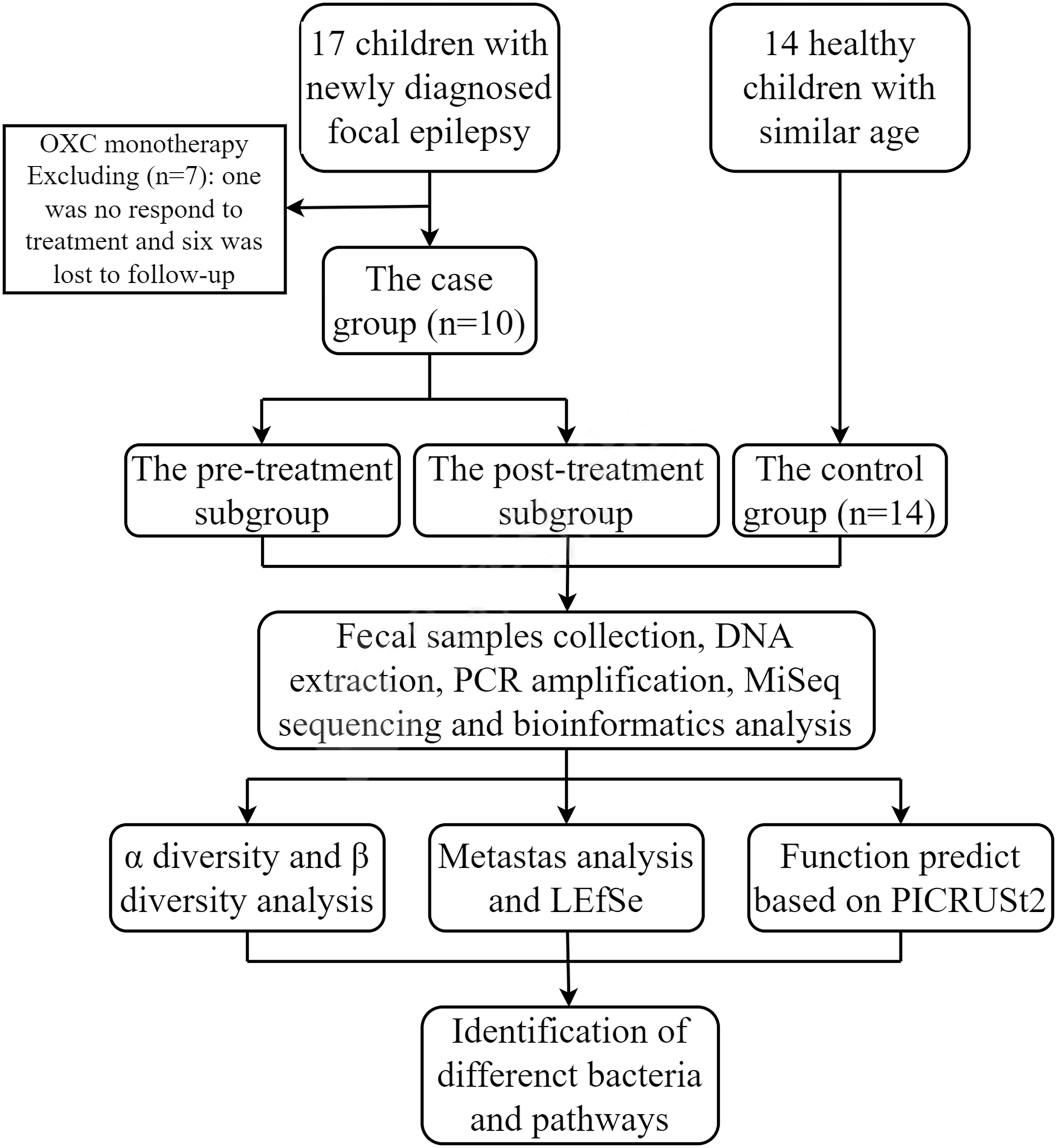
Flow diagram of the study.

We also recruited 14 age-similar, mentally and physically healthy children as a control group, who were not on any medications. Besides, the age of all subjects was more than three years old to avoid the confounding effect (Tanaka and Nakayama, 2017; Stewart et al., 2018). The recruitment process of all the subjects was shown in **Figure 1**.

The study was approved by the Ethics Committee of Hunan Children’s Hospital (No. HCHLL-2020-53). Written informed consent was obtained from the parents and/or legal guardians of the enrolled children.

### Clinical data and dietary data collection

Questionnaires were distributed to collect children’s baseline and follow-up information. The hospitalization data of children was extracted from the medical record system. The frequency, form, and duration of seizures were taken into consideration. Additionally, we collected the dietary data during treatment through online questionnaires, including the staple food and other types. Furthermore, “High”, “Moderate”, and “Low” three ranks were used to describe the intake frequency of vegetables and fruit, fried food and carbonated soft drinks, and milk.

### Clinical efficacy evaluation

The therapeutic effect was evaluated after using 3 months of OXC monotherapy. Seizure-free: There were no seizures during treatment. Significant reduction in seizures: The frequency of seizures was reduced by more than 50%. No change or increase in seizures: The frequency of seizures was reduced by less than 50% or increased. The total effective rate is equal to the control rate plus the effective control rate (Chen et al., 2014).

### Fecal samples collection

Fecal samples before treatment were collected and then stored at -80°C within half an hour. Fecal samples collected 3 months after the OXC monotherapy treatment were collected by their parents using a stool collection kit provided by Shanghai Genesky Biotechnology Inc., in which samples were stored for 2 weeks at room temperature, before being sent to the hospital and stored at -80°C.

### DNA extraction and high-throughput 16S rDNA gene sequencing

16S rDNA amplicon sequencing was performed by Genesky Biotechnologies Inc., Shanghai, 201315 (China). Briefly, total genomic DNA was extracted using the FastDNA^®^ SPIN Kit for Soil (MP Biomedicals, Santa Ana, CA) according to the manufacturer’s instructions. The integrity of genomic DNA was detected through agarose gel electrophoresis and the concentration and purity of genomic DNA were detected through the Nanodrop 2000 and Qubit 3.0 Spectrophotometer. The V4-V5 hypervariable regions of the 16S rDNA gene were amplified with the primers 515F (5’- GTGCCAGCMGCCGCGG -3’) and 907R (5’- CCGTCAATTCMTTTR AGTTT -3’) (Xiong et al., 2012) and then sequenced using Illumina NovaSeq 6000 platform.

The sequencing data were deposited to NCBI’s Sequencing Read Archive with the accession ID PRJNA846872.

### Gut microbial analysis

The raw read sequences were processed in QIIME2 (Bolyen et al., 2019). The adaptor and primer sequences were trimmed using the cutadapt plugin. DADA2 plugin was used for quality control and to identify amplicon sequence variants (ASVs) (Callahan et al., 2016). Taxonomic assignments of ASV representative sequences were performed with a confidence threshold of 0.8 by a pre-trained Naive Bayes classifier, which was trained on the RDP (version 11.5).

Curve analysis including the rarefaction curve, Shannon-Wiener curve, and species accumulation curve was performed to reflect the adequacy and rationality of the sample size. α diversity, evaluated by richness (Chao1 and ACE) and diversity (Shannon and Simpson). β diversity, based on Bray-Curtis distance, was calculated with QIIME2 and visualized with R (Version 4.1.3). The gene functions of the gut microbiota and the 16S rDNA gene sequences in the Kyoto Encyclopedia of Genes and Genomes (KEGG) were predicted by Phylogenetic Investigation of Communities by Reconstruction of Unobserved States 2 (PICRUSt2). The ANOVA and t-test were carried out appropriately for the comparison of overall differences between the groups.

Finally, we analyzed the microbial differences among groups at the phylum and genus levels using Metastats analysis (White et al., 2009) and linear discriminant analysis (LDA) effect size (LEfSe) (Segata et al., 2011).

### Statistical analysis

The general clinical information of the case and control groups was analyzed by SPSS. Shapiro-Wilk tests were performed to determine the normality of the data distribution. Continuous variables were expressed as mean with standard deviations or medians with interquartile ranges. Between-group differences were tested with the Mann-Whitney U test. For categorical variables, counts and percentages were presented. Between-group comparisons in categorical variables were tested with the χ2 test. *P*-value<0.05 was considered as statistically significant.

For microbial analysis, *P*-value<0.05 was considered statistically significant for simple, independent comparisons. However, for all analyses regarding multiple comparisons, we used the Benjamini–Hochberg method to correct *P*-value (FDR) for multiple testing (Benjamini and Hochberg, 1995). For comparisons of group differences, the Mann-Whitney U test, or Kruskal–Wallis test was used, where appropriate. Differences in metabolism among the three groups and within each group were identified through ANOVA and t-test.

## Results

### Characteristics of the study sample

There were no significant differences in age, gender, BMI, the dietary data between the case group and the control group (*P*>0.05, **Table 1**), and The staple food of all the subjects was grains. We followed the children in the case group for 3 months, during which the dose of oxcarbazepine was gradually increased to between 15 mg/kg/d and 30 mg/kg/d, and the treatment dose of the children was close to or at the maintenance dose of the drug (20 mg/kg/d to 30 mg/kg/d). No obvious adverse reactions such as rash, diarrhea, liver or kidney function impairment were reported. Furthermore, during treatment, seven cases did not have a recurrence of epilepsy and the frequency of seizures in three cases was significantly lower than before. The EEG was not reviewed during the follow-up period. The follow-up data were presented in **Supplementary Table 1**.

**Table 1:** Comparison of clinical data between 2 groups.

Characteristics	Case group (n = 10)	Control group (n = 14)	Z/χ^2^	*P*-value
Age (year)^a^	6.35 (5.40, 9.50)	5.15 (3.98, 7.90)	-1.906	0.057
Gender (males, %) ^b^	5 (50.00)	5 (50.00)	0.120	1.000
BMI (kg/m2) ^a^	14.41 (13.58, 15.90)	15.22 (14.55, 15.62)	-0.586	0.558
Dietray data (n, %) ^a^				
Vegetables, and fruit			-0.392	0.752
High	4 (40.00)	8 (57.14)		
Moderate	6 (60.00)	4 (28.57)		
Low	0	2 (14.29)		
Fried food, and carbonated soft drinks			-0.564	0.666
High	1 (10.00)	1 (7.14)		
Moderate	3 (30.00)	3 (21.43)		
Low	6 (60.00)	10 (71.43)		
Milk			-2.082	0.064
High	3 (30.00)	11 (78.57)		
Moderate	4 (40.00)	1 (7.14)		
Low	3 (30.00)	2 (14.29)		

Data are expressed as mean (standard error of mean) for continuous variables or n (percentage) for categorical variables.

Differences between the two groups were analyzed using the Mann-Whitney U test (marked as ^a^) for quantitative variables. χ^2^ test was used for categorical variables (marked as ^b^).

### Gut microbial diversity and relative abundance of fecal bacterial community

**Figure 2:**
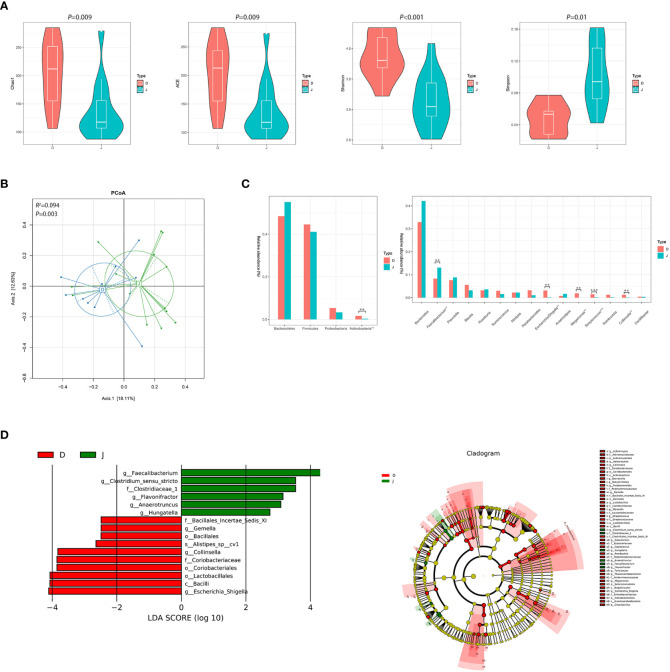
Diversity and relative abundance of gut microbiota between the group before treatment and the control group. **(A)** Comparison of the α diversity indices between the two groups. **(B)** PCoA between the two groups based on Bray-Curtis distance. **(C)** Histogram showing the top 4 phyla (the left) and the top 15 genera (the right) by abundance. **FDR<0.01, ***FDR<0.001. **(D)** LEfSe with regard to abundance. D: pre-treatment group, J: control group.

There were significant differences between the pre-treatment subgroup and the control group in terms of α diversity. Specifically, Chao1, ACE, and Shannon index were higher and Simpson index was lower in the pre-treatment subgroup (**Figure 2A**, *P*<0.05). PCoA also found different microbial communities between these two groups (ADONIS, *R^2 =^* 0.094, *P*=0.003, **Figure 2B**). Metastats analysis identified that at the phyla level, the relative abundance of *Actinobacteria* was significantly higher in the pre-treatment subgroup than in the control group (0.016 *vs.* 0.003, *FDR*=0.035). At the genus level, *Escherichia/Shigella*, *Streptococcus*, *Collinsella* and *Megamonas* in the pre-treatment subgroup were enriched (0.031 *vs.* 0.002, *FDR*=0.016; 0.015 *vs.* 0.002, *FDR*=0.009; 0.012 *vs.* 0.001, *FDR*=0.021; 0.019 *vs.* 0.001, *FDR*=0.021, respectively), while *Faecalibacterium* was enriched in the control group (0.083 *vs.* 0.131, *FDR*=0.043; **Figure 2C** and **Supplementary Figure 1**). In addition, LEfSe also revealed a reduction of *Faecalibacterium*, along with a growth of *Escherichia/Shigella* and *Collinsella* (**Figure 2D**) in the pre-treatment subgroup.

**Figure 3:**
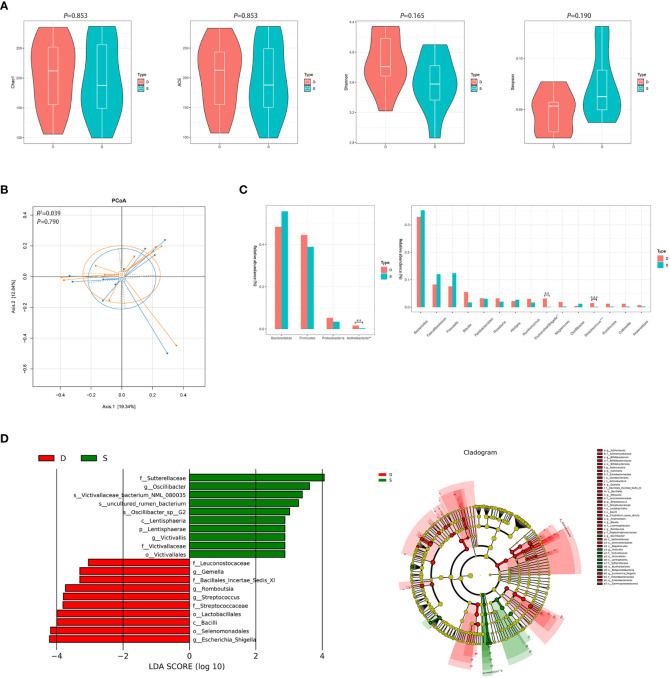
Diversity and relative abundance of gut microbiota between the group before and after treatment. **(A)** Comparison of the α diversity indices between the two groups. **(B)** PCoA between the two groups based on Bray-Curtis distance. **(C)** Histogram showing the top 4 phyla (the left) and the top 15 genera (the right) by abundance. **FDR<0.01, ***FDR<0.001. **(D)** LEfSe with regard to abundance. D: pre-treatment group, S: post-treatment group.

After 3 months of monotherapy treatment, no significant differences were found in α diversity compared between the pre-treatment subgroup and the post-treatment subgroup (**Figure 3A**). PCoA failed to reveal the different composition between the two subgroups (ADONIS, *R^2 =^* 0.039, *P*=0.790, **Figure 3B**). Metastats analysis identified that at the phyla level, the relative abundance of *Actinobacteria* after treatment obviously declined compared to the pre-treatment subgroup (0.016 *vs.* 0.003, *FDR*=0.015). At the genus level, the proportion of some genera decreased significantly, such as *Escherichia/Shigella* and *Streptococcus* (0.031 *vs.* 0.001, *FDR*=0.022; 0.015 *vs.* 0.002, *FDR*=0.012, respectively) (**Figure 3C** and **Supplementary Figure 2**). LEfSe also revealed a decrease in *Escherichia/Shigella* and *Streptococcus* (**Figure 3D**) in the post-treatment subgroup.

**Figure 4:**
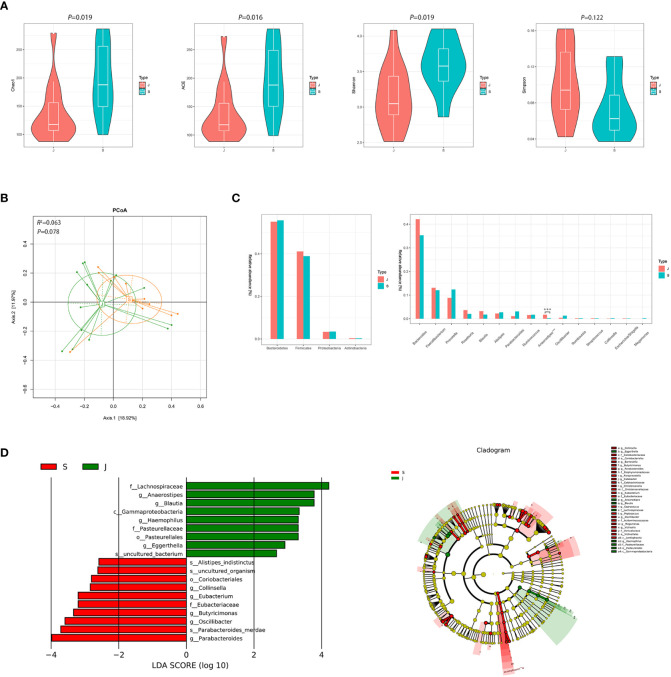
Diversity and relative abundance of gut microbiota between the group after treatment and the control group. **(A)** Comparison of the α diversity indices between the two groups. **(B)** PCoA between the two groups based on Bray-Curtis distance. **(C)** Histogram showing the top 4 phyla (the left) and the top 15 genera (the right) by abundance. ***FDR<0.001. **(D)** LEfSe with regard to abundance. S: post-treatment group, J: control group.

Despite significant differences in α diversity (**Figure 4A**), the differences in bacterial composition between the post-treatment subgroup and control group were not significant (ADONIS, *R^2 =^* 0.063, *P*=0.078, **Figure 4B**). Moreover, Metastats analysis suggested a decrease in the number of significantly different genera that were compared between the post-treatment subgroup and control group (**Figure 4C** and **Supplementary Figure 3**). Importantly, the differences in the relative abundance of *Escherichia/Shigella* and *Streptococcus* were not significant. Nevertheless, the relative abundance of *Anaerostipes* was lower in the post-treatment subgroup group compared to the control group (0.003 *vs.* 0.017, *FDR*=0.013). LEfSe between the two groups found the same results (**Figure 4D**).

**Figure 5:**
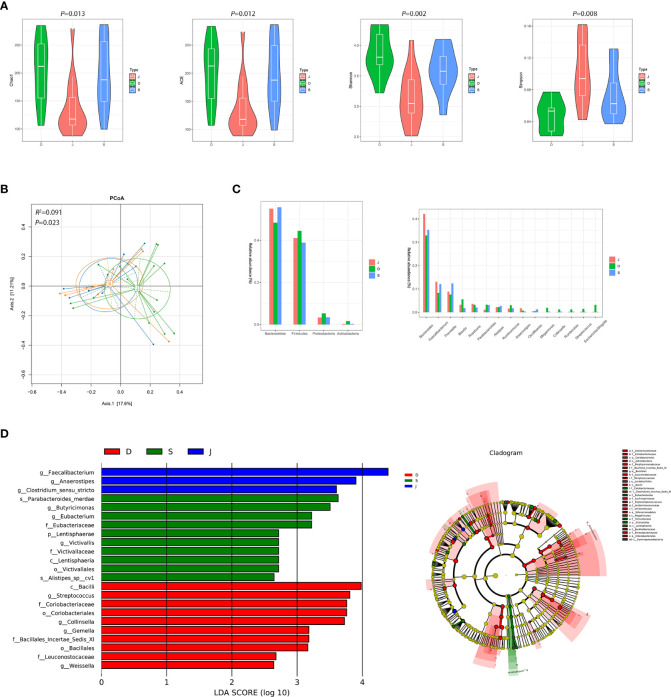
Diversity and relative abundance of gut microbiota among the pre-treatment group, post-treatment group, and the control group. **(A)** Comparison of the α diversity indices between the three groups. **(B)** PCoA among the three groups based on Bray-Curtis distance. **(C)** Histogram showing the top 4 phyla (the left) and the top 15 genera (the right) by abundance. **(D)** LEfSe with regard to abundance. D: pre-treatment group, S: post-treatment group, J: control group.

Although there were significant differences in α diversity among the three groups, a trend of alpha diversity decreasing with treatment was still observed (**Figure 5A**). PCoA revealed that the microbial communities among the three groups was significantly different (ADONIS, *R^2 =^* 0.091, *P*=0.023, **Figure 5B**). At the phyla level, the gut microbiota in the three groups was mainly composed of *Bacteroidetes* and *Firmicutes*. At the genus level, the gut microbiota was dominated by *Bacteroides* (**Figure 5C**). LEfSe among the three groups showed that the gut microbiota of the pre-treatment subgroup was mainly dominated by *Streptococcus* and *Collinsella*, the post-treatment subgroup was mainly dominated by *s_Parabacteroides_merdae*, and the control group was mainly dominated by *Faecalibacterium* and *Anaerostipes* (**Figure 5D**). Furthermore, changes in the relative abundance of some bacteria in **Figure 5C** suggested that they were related to focal epilepsy, such as *Faecalibacterium*, *Anaerostipes*, *Escherichia/Shigella*, *Streptococcus*, *Collinsella*, and *Megamonas*.

### KEGG-based comparison of metabolic pathways among the three groups

**Figure 6:**
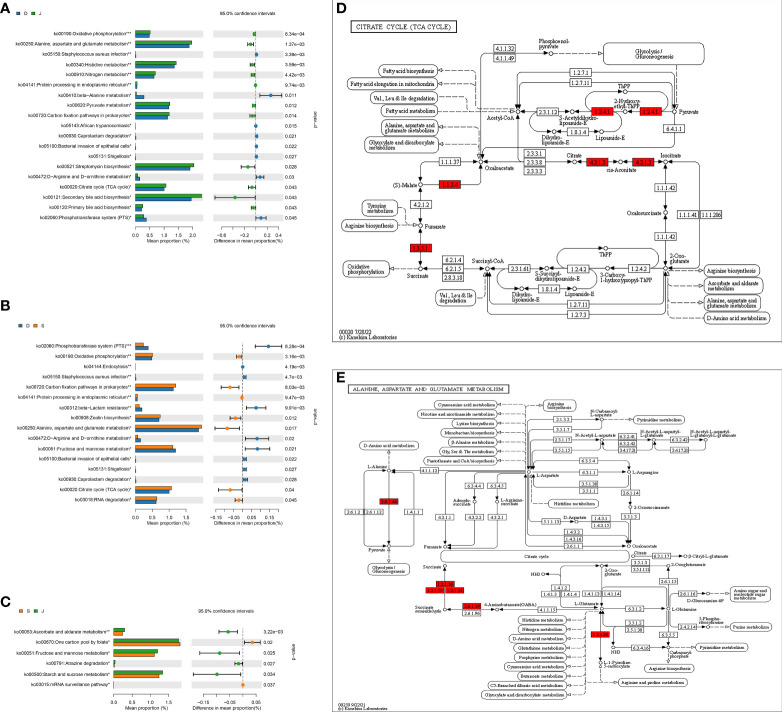
level 3 Metabolic pathway analysis based on a pairwise comparison among the three groups. **(A)** Comparison between the pre-treatment subgroup and the control group. **(B)** Comparison between the pre-treatment subgroup and the post-treatment subgroup. **(C)** Comparison between the post-treatment subgroup and the control subgroup. **(D, E)** Differences in alanine, aspartate, glutamate metabolism, and differences in citrate cycle (TCA cycle), respectively. *FDR<0.05, **FDR<0.01, ***FDR<0.001. Red: upregulation. D: pre-treatment group, S: post-treatment group, J: control group.

ANOVA was used to determine global metabolic differences in 3^rd^-level KEGG pathways, and 21 significantly different 3^rd^-level pathways were identified. The t-test revealed significant differences in 19 pathways between the pre-treatment subgroup and the control group, 16 pathways between the pre-treatment subgroup and the post-treatment subgroup, and 6 pathways between the post-treatment subgroup and the control group (**Figures 6A–C**). We identified that the pathways associated with alanine, aspartate, glutamate metabolism, and citrate cycle (TCA cycle) contributed to the differences mostly among the three groups. Children in the pre-treatment subgroup showed upregulation of certain genes (**Figures 6D, E**).

## Discussion

In the present study, we found α diversity of the pre-treatment subgroup was significantly higher than that of the control group. Even α diversity still significantly differed from healthy children after 3-month treatment, the diversity showed an overall downward trend. Besides, PCoA identified a different microbial community between the pre-treatment subgroup and the control group. A previous study found that the α diversity of the drug-resistant epilepsy group was significantly higher than that of the drug-sensitive epilepsy group and the healthy control group, and α diversity between the latter two groups was close (Peng et al., 2018). Zhang and teammates suggested that the diversity in children with intractable epilepsy decreased after 6 months of KD treatment, and observed that seizures were effectively controlled in 10 children (Zhang et al., 2018). Furthermore, Carlson and colleagues revealed that the diversity of gut microbiota was associated with cognitive function, as the higher the α diversity was, the worse cognitive performance would be (Carlson et al., 2018). Therefore, we thought that compared with healthy children, children with focal epilepsy had higher α diversity and different microbial communities, which could be recovered *via* effective treatment. However, there was no statistical significance in the decrease of α diversity between the pre- and post-treatment subgroups, or PCoA failed to show the different microbiota structure between the pre- and post-treatment subgroups, which was related to the course of treatment and even the type of antiepileptic drugs.

Our study also showed that the gut microbiota in the 3 groups was mainly composed of *Bacteroidetes* and *Firmicutes* at the phylum level. Previous studies have shown that the gut microbiota of healthy children after 12 months tends to be like that of adults, dominated by *Bacteroidetes* and *Firmicutes*. Our findings were consistent with these, indicating that children with focal epilepsy have the same type of gut microbiota as healthy children (Palmer et al., 2007; Zheng et al., 2017), yet differences in abundance were observed. Interestingly, after effective treatment, the difference in microbial communities between the post-subgroup and the control group disappeared. A study found that the gut microbial communities of the drug-sensitive epilepsy group was similar to that of the healthy control group, mainly dominated by *Bacteroides* and *Firmicutes* (Peng et al., 2018), suggesting an association between changes in gut microbiota and epilepsy efficacy.

Our results revealed that some agreement with the majority of the data that are currently published. Several studies found after KD treatment, children with intractable epilepsy had relieved clinical symptoms and the relative abundance of *Bacteroidetes* increased while *Actinobacteria* decreased (Zheng et al., 2017; Lindefeldt et al., 2019). We also found a significant decrease in *Actinobacteria* after OXC treatment, in line with the earlier studies. However, one existing study showed that the abundance of *Bacteroidetes* and *Actinobacteria* in the healthy group was higher than those in the epilepsy group (Şafak et al., 2020). This difference might be due to the possible fact that the study did not account for the curative effect on the abundance of *Actinobacteria* in children with epilepsy, or other related factors such as race and lifestyle.

In addition to the different relative abundance of *Actinobacteria*, combining Metastas analysis and LEfSe, we found that changes in the relative abundance of some genera could be associated with focal epilepsy. Specifically, the increase of *Escherichia/Shigella*, *Streptococcus*, *Collinsella*, and *Megamonas*, and the decrease of *Faecalibacterium* and *Anaerostipes* might be related to focal epilepsy. Their relative abundance was closer to that of healthy children after treatment, which might be associated with clinical remission of focal epilepsy.

*Escherichia/Shigella* was associated with pro-inflammatory status, and other studies showed that *Escherichia/Shigella* induced proinflammatory cytokines through NLRP3 inflammasome-dependent mechanisms (Morgan, 2013; De la Fuente et al., 2014). Additionally, previous studies found that the abundance of pro-inflammatory *Escherichia/Shigella* in stool was higher in patients with cognitive impairment and brain amyloidosis than in the control group, and was positively related to the level of IL-1β, CXCL2 and NLRP3 inflammasome (Cattaneo et al., 2017). There were many studies about inflammasome and how it was involved in the development of neurological diseases such as Alzheimer’s disease, multiple sclerosis, stroke and traumatic brain injury (Heneka et al., 2018; Voet et al., 2019). In addition, melatonin has been proven to reduce the incidence of seizures and epilepsy (Aydin et al., 2015). Moreover, according to building an epileptic mouse model and treating it with melatonin, Jia’s team found that NLRP3, caspase-1 and IL-1β increased significantly in untreated epileptic mice compared with treatment and control groups (Jia et al., 2018). These results suggested that *Escherichia/Shigella* might be involved in the occurrence and development of epilepsy through inflammatory mechanisms. Our results also showed *Streptococcus*, notorious pathogenic bacteria, enriched in the pre-treatment group. According to some studies, enrichment of *Streptococcus* could increase the level of IL-6 and TNF-α (Jiang et al., 2015). TNF-α could increase nervous system excitability *via* Ca^2+^ influx, and IL-6 was correlated with epileptic seizures (Galic et al., 2012; Rana & Musto, 2018). Thus, *Streptococcus* might be involved in epilepsy through neuroinflammation in children with focal epilepsy.

Our results showed *Collinsella* and *Megamonas* in the pre-treatment subgroup were significantly higher than those in the control group, which reduced distinctly after treatment. Gut microbiome-brain signaling was linked with different signaling factors, in which short-chain fatty acids (SCFAs) played an important role (Erny et al., 2015; Dalile et al., 2019). Some previous studies found the enrichment of *Collinsella* could inhibit the growth of fermenting bacteria producing SCFAs (Candela et al., 2016; Gomez-Arango et al., 2018). Moreover, *Collinsella* could increase intestinal permeability by reducing the expression of tight junction proteins in epithelial cells and inducing the expression of IL-17 which was found to be at a higher level in patients with epilepsy. The inter-seizure IL-17A levels were positively correlated with the frequency and severity of seizures (Mao et al., 2013; Chen et al., 2016). One study showed that *Megamonas* had a significant increase in children with autism spectrum disorder (Zou et al., 2020). Therefore, *Collinsella* might play a detrimental role in children with focal epilepsy, while the significance of *Megamonas* in children with focal epilepsy, even different types of epilepsy, needs to be further studied.

We found *Faecalibacterium* and *Anaerostipes* were enriched in the control group compared with the group before and after treatment, indicating that its abundance decrease might be related to focal epilepsy. *Faecalibacterium* was a class of bacteria that could produce SCFAs (Joseph et al., 2017). In addition to the above-mentioned roles, SCFAs could also reduce the permeability of the blood-brain barrier in animal experiments by upregulating the expression of tightly linked proteins (Braniste et al., 2014). Moreover, it has been reported that injection of physiological concentrations of SCFAs in the proximal colonic lumen of rats stimulated the release of 5-hydroxytryptamine (5-HT) by intestinal chromophiles, and then 5-HT7 acted as a receptor for 5-HT, which could inhibit seizures after activation (Fukumoto et al., 2003; Pericić & Svob Strac, 2007). *Anaerostipes* could translate propionate to butyrate, which has been confirmed to have the ability to be antidepressant and anti-inflammatory *via* inactivating the microglia in the mouse model (Yamawaki et al., 2018). Therefore, *Faecalibacterium* and *Anaerostipes* may be involved in the mechanisms of epilepsy and even more neurological disorders through the SCFAs pathways.

We also identified the pathways associated with alanine, aspartate, glutamate metabolism and citrate cycle (TCA cycle) were significantly different among the three groups, especially between the pre-treatment subgroup and control group. Moreover, the gene upregulated seemed to promote the production of succinate, an intermediate of TCA cycle, and it was demonstrated that succinate accumulation likely plays a vital role in kainic acid-induced status epilepticus in a rat model (Zhang et al., 2020). A study based on a chronic pilocarpine mouse epilepsy model also found that fructose 1,6-bisphosphate worked to alleviate epilepsy and improved elements of glucose metabolism, accompanied by the reduction of succinate and others in the hippocampal formation (McDonald et al., 2021). Therefore, we thought that apart from the abovementioned mechanisms, the gut microbiota might exert effects on epilepsy through carbohydrate metabolism, and the potential target was succinate.

There were some limitations in our study. Firstly, this study was a single-center study with small sample size, and the methods we used were associative, not causative or experimental. Therefore, the causal association between the gut microbiota and focal epilepsy was difficult to determine. Secondly, the relationship between gut microbiota and the therapeutic effect of epilepsy was not fully understood due to a lack of comparison with a group that did not respond to treatment. Finally, this study had a short follow-up and sampled twice, which was unable to obtain the dynamics of the gut microbiota during treatment. However, our study was the first to select newly diagnosed children who did not take antiepileptic drugs as research subjects. We selected children over 3 years of age to avoid age-related interference with gut microbiota, and the effects of probiotics, antibiotics, and weight on gut microbiota were excluded in the study.

## Conclusion

Compared with the control group, the relative abundance of *Faecalibacterium*, *Collinsella*, *Escherichia/Shigella*, *Streptococcus* and *Megamonas* was observed to the significant difference in patients with focal epilepsy. Besides, there were significant differences in microbial communities before and after treatment, and gut microbiota of those with effective treatment changed to healthy children, indicating that therapeutic effect may be related to the changes of gut microbiota. The differences were reduced and the carbohydrate metabolism improved after effective monotherapy.

## Data availability statement

The data presented in the study are deposited in the National Library of Medicine (NCBI) repository, accession number PRJNA846872.

## Ethics statement

The studies involving human participants were reviewed and approved by the Ethics Committee of Hunan Children’s Hospital (Changsha, China. No. HCHLL-2020-53). The parents/guardians of participating children provided their written informed consent to participate in this study.

## Author contributions

SZG, CCZ and JQ contributed to the conception and design of the work. RWH, ZYL, ZHX and JQ provided administrative support. LJL, XH, YM and ZHX provisioned the study materials or patients. SZG, LJL, XH and YM collected and assembled the data. CCZ, STX and JQ performed the statistical analyses and interpretated. All authors contributed to the manuscript writing, and all authors read and approved the final version of manuscript. All authors contributed to the article and approved the submitted version.

## Funding

This research was funded by the Science and Technology Department of Hunan Province (2017SK2154, and 2020SK1014-3).

## Acknowledgments

The authors thank all the participant children and their parents in the study and appreciate all of the support of the data collectors. All authors had access to the study data and reviewed and approved the final manuscript.

## Conflict of interest

The authors declare that the research was conducted in the absence of any commercial or financial relationships that could be construed as a potential conflict of interest.

## Publisher’s note

All claims expressed in this article are solely those of the authors and do not necessarily represent those of their affiliated organizations, or those of the publisher, the editors and the reviewers. Any product that may be evaluated in this article, or claim that may be made by its manufacturer, is not guaranteed or endorsed by the publisher.
