# Supplementary material for: Changes and significance of gut microbiota in children with focal epilepsy before and after treatment
Source: Front Cell Infect Microbiol. 2022 Nov 3;12:965471. doi: 10.3389/fcimb.2022.965471 (PMC9671114; doi:10.3389/fcimb.2022.965471)
Supplement: Supplementary file 1 [file DataSheet_1.docx]

Supplementary Material

# Supplementary Figures and Tables

## Supplementary Tables

Supplementary Table 1. Follow-up data from 10 children with focal epilepsy.

| Number | Course | EEG | Head MRI | Frequency of seizure before treatment | Frequency of seizure during treatment | Adverse reaction |
| --- | --- | --- | --- | --- | --- | --- |
| 1 | 3 months | The sharp waves in the right temporal region fired in a slightly more amount, and the amplitude in the middle temporal region was the highest. | Normal | 2 times/3 months | No seizure | No |
| 2 | 1 year | Middle and posterior temporal region on the right δ activity and sharp waves fired in a small amount, and one focal seizure was detected. | Normal | 2-3 times per day 1 month before treatment, lasting about 1 minute | Occasional seizures at the beginning of treatment, lasting half a minute, and no seizure since the second month of treatment | No |
| 3 | 3 years | Spike waves fired occasionally in the left frontal pole, frontal and anterior temporal regions. | Normal | 2 seizures occurred 3 days before treatment | No seizure | No |
| 4 | 2 years | A large number of sharp waves and sharp slow waves fired in bilateral central and parietal temporal regions. | Normal | 2 seizures occurred 3 months before treatment, lasting 5-6 minutes | 1 seizure during treatment, lasting 1 minute | No |
| 5 | Half one month | Sharp waves and sharp slow waves fired in Rolandic region. | Normal | 8 times/ half one month | No seizure | No |
| 6 | 3 months | A large number of sharp waves and sharp slow waves fired in Rolandic region. | Normal | 2 times/3 months | No seizure | Itching occasionally |
| 7 | 1 month | Slow waves in the right temporal region fired in a slightly more amount, and sharp waves and sharp slow waves in the apical, temporal regions (right temporal region) and the apical midline fired in a slightly more amount. | The left lateral ventricle widened to the greater occipital cistern | 2 times per month | No seizure | No |
| 8 | 1 year | Sharp waves and sharp slow waves in Rolandic region fired in a slightly more amount, and 2 focal seizures were detected. | Normal | 1 time per day 1 week before treatment, lasting more than 10 seconds | 3 seizures during treatment, lasting several seconds | No |
| 9 | 10 months | Sharp waves and sharp slow waves in Rolandic region (right) fire in a slightly more amount. | The temporal angle of the left lateral ventricle was slightly larger and deformed than that of the opposite side | 1 time/ month | No seizure | No |
| 10 | 2 months | Slow waves in the front head fire in a slightly more amount, and anterior cephalic or generalized spike slow wave dispersion | Normal | 3 times/2 months | No seizure | No |

## Supplementary Figures


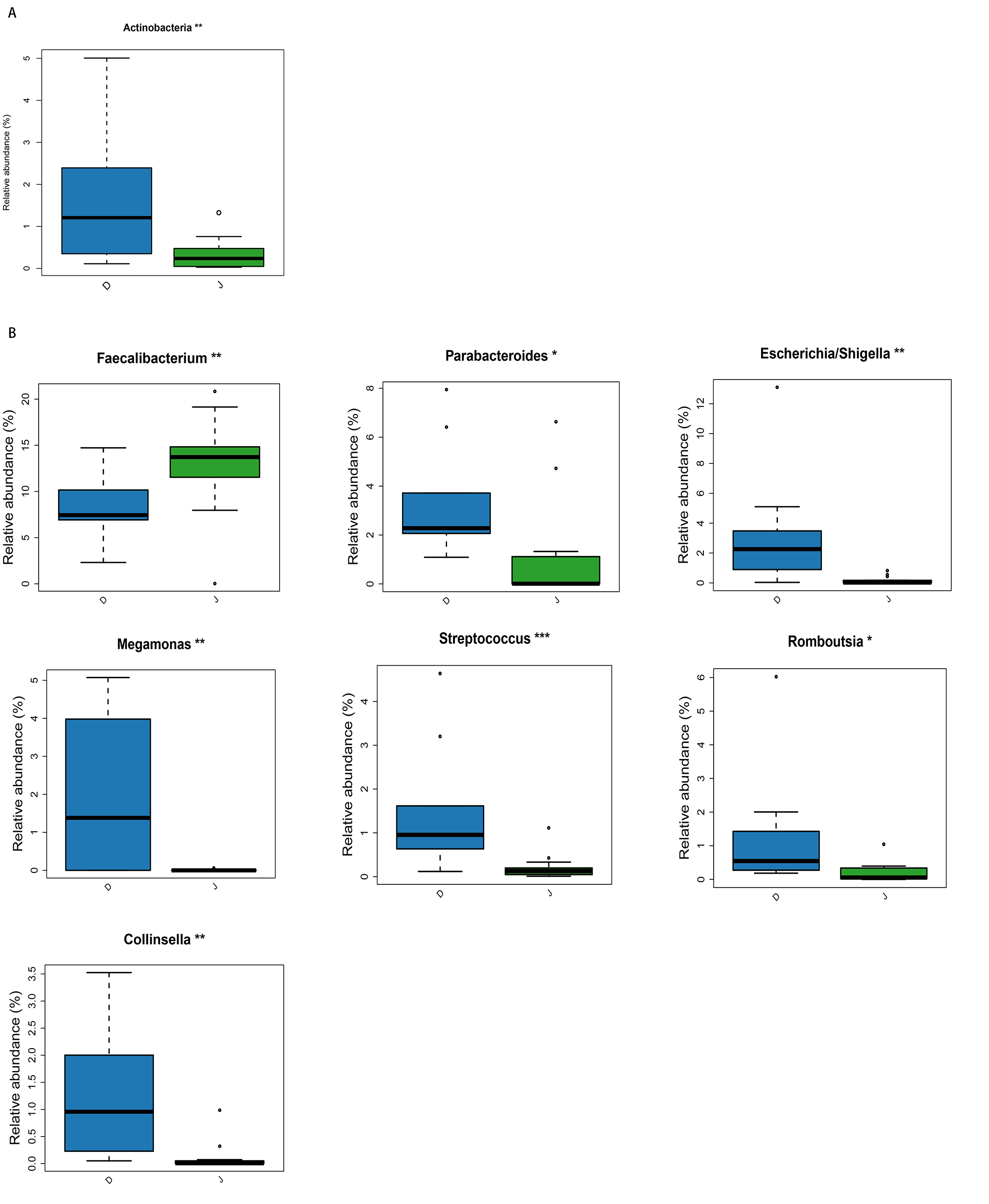


**Supplementary Figure 1.** Significant results of Metastats analysis related to Figure 2. (A) At the phyla level, and (B) At the genus level.

**
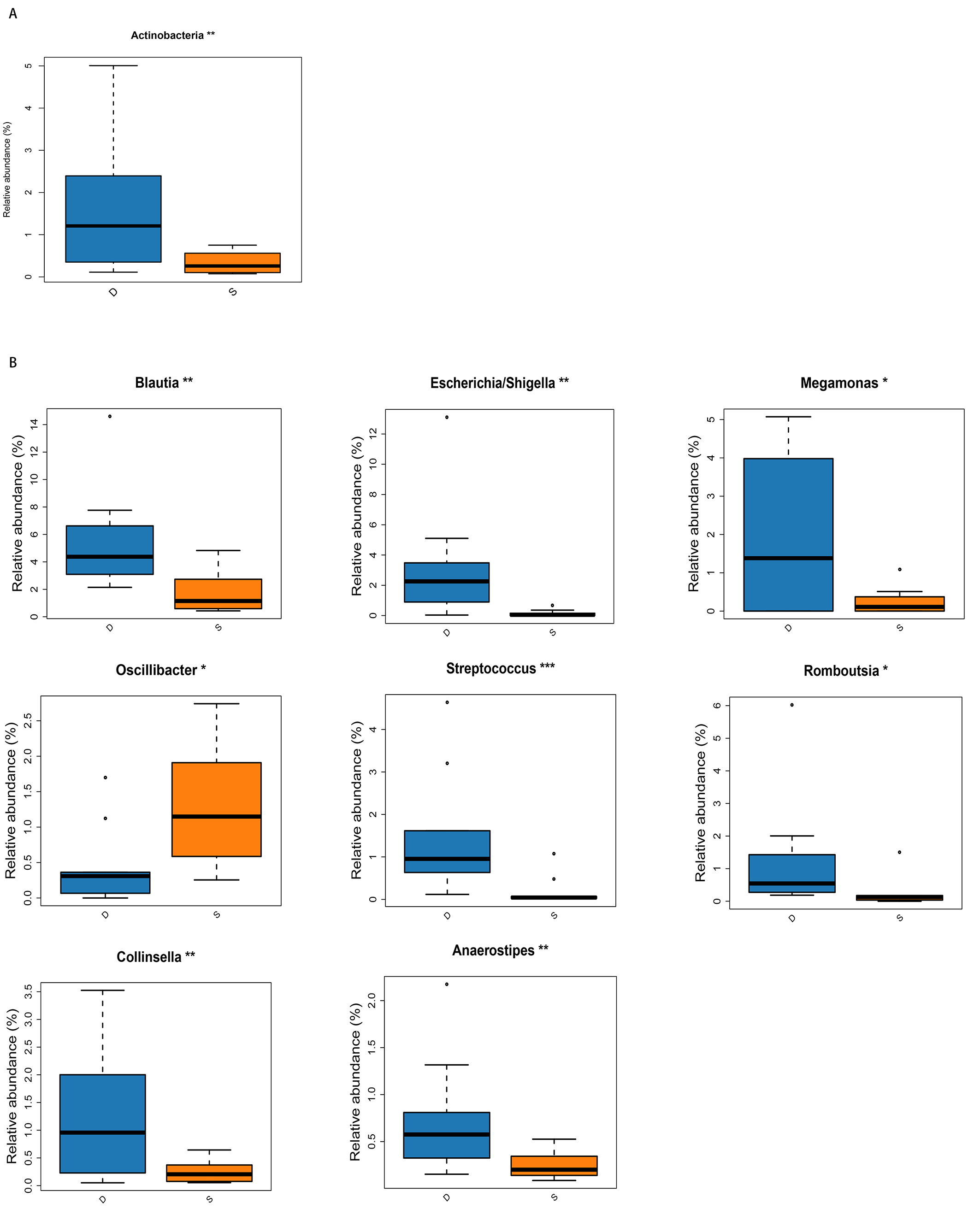
**

**Supplementary Figure 2.** Significant results of Metastats analysis related to Figure 3. (A) At the phyla level, and (B) At the genus level.

**
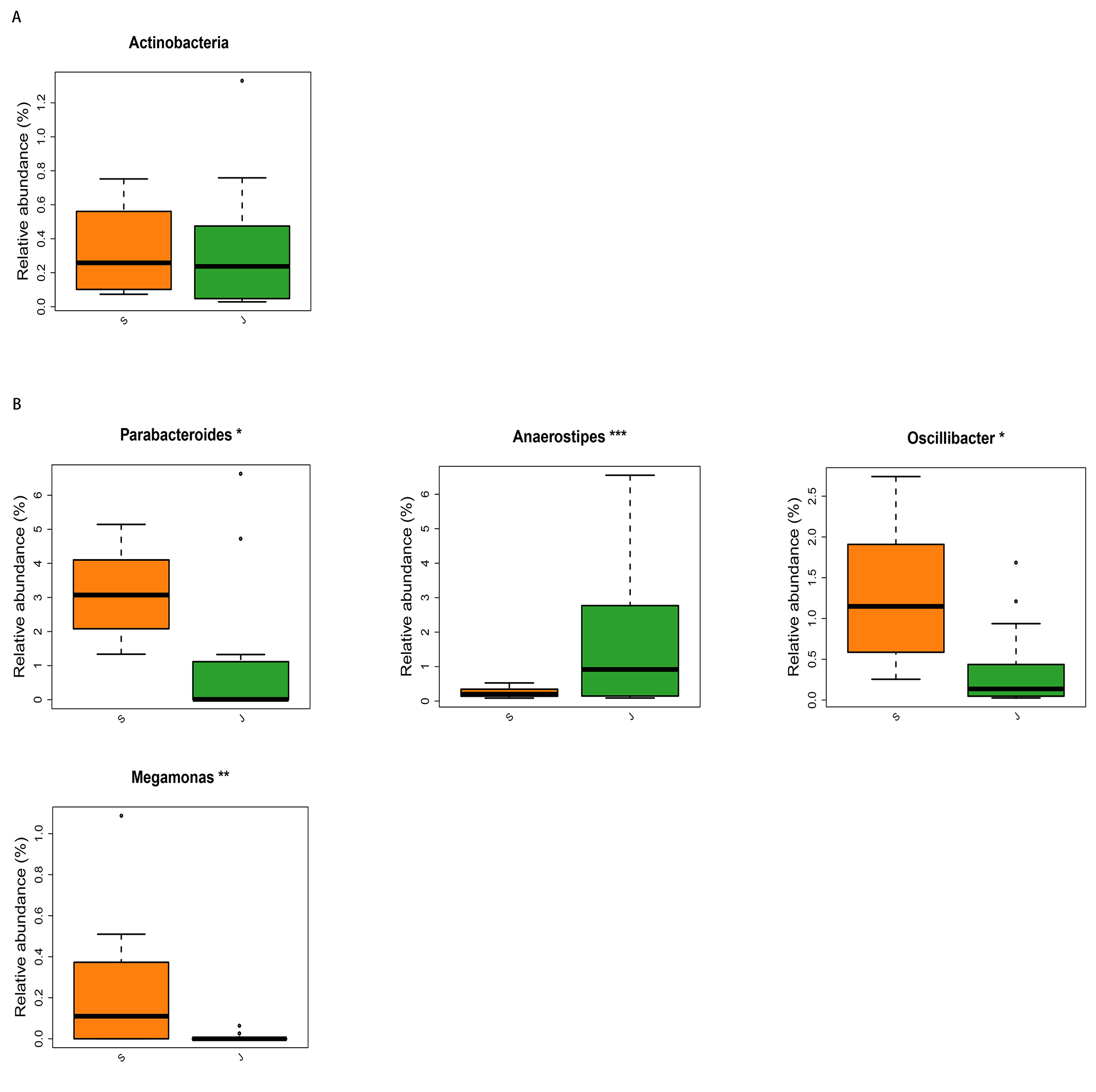
**

**Supplementary Figure 3.** Significant results of Metastats analysis related to Figure 4. (A) At the phyla level, and (B) At the genus level.
